# An Overview of Developmental Dysplasia of the Hip and Its Management Timing and Approaches

**DOI:** 10.7759/cureus.45503

**Published:** 2023-09-18

**Authors:** Ali Alhaddad, Amin G Gronfula, Thamer H Alsharif, Ahmed A Khawjah, Mohammed Y Alali, Khalid M Jawad

**Affiliations:** 1 Orthopedic Surgery, East Jeddah General Hospital, Jeddah, SAU; 2 Orthopaedic Surgery, Royal College of Surgeons in Ireland, Dublin, IRL; 3 Neurosurgery, Royal College of Surgeons in Ireland, Dublin, IRL; 4 Emergency Medicine, Royal College of Surgeons in Ireland, Dublin, IRL; 5 Orthopaedics, East Jeddah General Hospital, Jeddah, SAU

**Keywords:** ddh management, paediatric orthopedic, hip dysplasia, congenital dislocation of the hip, ddh

## Abstract

Developmental dysplasia of the hip (DDH), if uncorrected, can result in several chronic abnormalities, including chronic hip pain, degenerative arthritis, and gait abnormalities. The outcome of DDH generally depends on the age of presentation; a worse prognosis is linked to a higher age of presentation. Although treatment continues to be a challenge, recent advancements in the field have improved our understanding of the disease, which has resulted in advancements in DDH surveillance during infancy and the reduction of complications with early intervention. The databases used for this overview include Cochrane Central Register of Controlled Trials (CENTRAL), the Cochrane Library, MEDLINE, and EMBASE. These databases were used to search for ongoing trials related to the management and diagnosis of DDH.

## Introduction and background

Developmental dysplasia of the hip (DDH) is a term that encompasses a variety of hip abnormalities; these abnormalities range from neonatal instability, acetabular dysplasia, hip subluxation, to true hip dislocation [1, 2]. Intervention and prognosis vary depending on the type of abnormality, which is why it is essential to differentiate between the different types of abnormalities in the DDH spectrum [3]. A total of 13 in 1,000 females and 5 in 1,000 males are reported to have hip instability, making up around 1-1.5% of newborns [4]. Within the first two months of life, approximately 90% of mild hip instabilities resolve spontaneously [5], while a small percentage of these persist as instabilities and/or echogenic changes. These persistent alterations are termed "persistent dysplasia." With persistent dysplasia, the biomechanics of the hips are altered, resulting in an increased load on the articular cartilage, which ultimately results in chronic complications that include chronic hip pain, degenerative arthritis, and gait abnormalities [6].

Although there is still debate on what is the ideal method of screening for DDH, the aim of screening remains the same, which is essentially to allow for early intervention and, as a result, aid in achieving hip reduction in the least aggressive method [5,6]. What makes screening for DDH challenging is the fact that there is no unifying pathological feature that defines DDH, as the term encompasses everything from mild hip dysplasia to true hip dislocation. Another factor is that the normal immature hip in neonates can share similar features to the pathological hip in DDH due to ligamentous laxity, which often spontaneously resolves. These features can include a positive Barlow examination or hip instability on ultrasonography [7].

## Review

Anatomical changes in dysplastic hip

Over time, numerous adaptive changes occur, affecting all components of the hip. For the acetabular cavity to develop correctly, it must be well-aligned with the femoral head. In situations where the femoral head is not fully aligned, the acetabular cavity tends to flatten. The labrum, capsule, and transverse ligament can hypertrophy. When the labrum becomes hypertrophied, it forms the limbus. The limbus may evert, which is more common, or invert, which hinders hip reduction.

It is crucial to differentiate between the limbus and the neolimbus. The neolimbus is a hypertrophied portion (ridge) of the acetabular cartilage, formed as a result of excessive pressure from the subluxated femoral head against the posterosuperior part of the acetabulum. When the neolimbus forms, the articular cavity bifurcates into the primary and secondary acetabulum. The primary is the medial zone, and the secondary is the lateral zone. When the hip is reduced, the neolimbus no longer persists [8]. Although it was traditionally believed that incidences of acetabular anteversion were higher in hip dysplasia, some studies have found no difference in acetabular anteversion occurrence rates between dysplastic and non-dysplastic hips [9, 10].

For the hip to grow and mature normally, it depends on two essential factors, and any deviations from these result in hip dysplasia. These factors are the appropriate alignment of the femoral head within the acetabular cavity and the parallel, simultaneous growth of the triradiate and acetabular cartilage [8]. Dunn et al. [11] observed no instances of DDH in fetuses that were aborted before 20 weeks of gestation, leading to the conclusion that most changes accounting for DDH occur during the later months of the fetus's intrauterine life.

Numerous theories have been proposed to explain the development of DDH; among them is the hormonal theory. This theory suggests that a mismatch between estrogen and progesterone may be the cause. Studies have indicated that estrogen can act as a protective hormone against hip dislocation, whereas higher levels of progesterone increase the risk of hip dislocation [12]. However, no correlation between DDH and serum levels of β-estradiol and relaxin was found. Gender seems to play a more significant role than hormones [12].

Diagnostic methods

Screening for DDH relies on clinical examination, ultrasound (US), or both. Screening can be targeted either at the general newborn population or at a high-risk cohort. While X-rays were used in the past, they are not commonly used today; hence, this overview will not delve into X-ray as a diagnostic method. The high-risk group includes newborns with a first-degree relative with DDH, breech presentation, being female, congenital torticollis, talipes, high birth weight, oligohydramnios, and metatarsus adductus [3, 6]. Racial disparities also exist in occurrence rates.

During examination, factors that may indicate DDH include limitations in hip abduction, thigh fold asymmetry, and limb length disparities. These are followed by Barlow's and Ortolani's tests. Barlow's test functions by dislocating the normally aligned but unstable femoral head, while Ortolani's test functions by realigning the dislocated femoral head. A positive test is noted if instability is felt or a "clunk" is heard during the test (whereas routinely felt clicks are considered insignificant) [9, 10, 12].

Imaging methods in the diagnosis of DDH

Radiography

**Figure 1:**
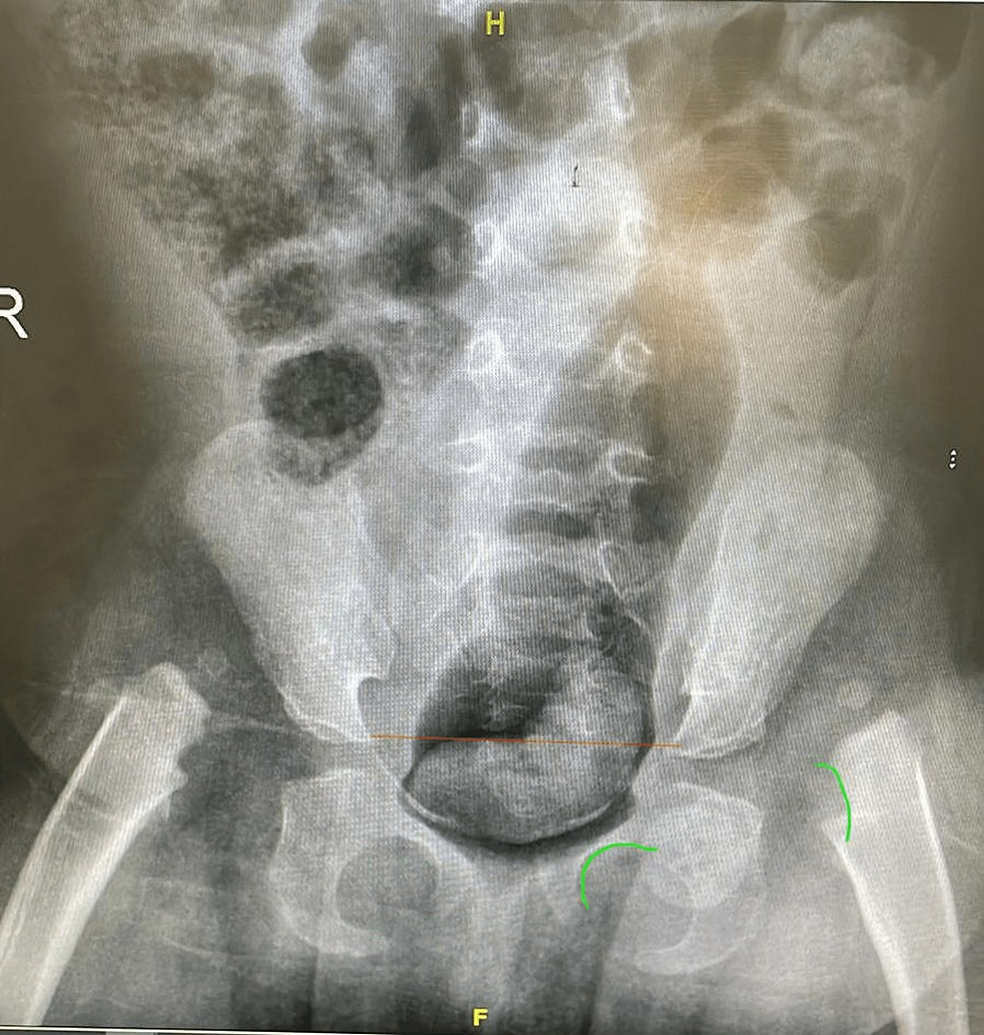
Shenton's line (green lines). The acetabular index (red line) is the main parameter to control acetabular development during the first years of age. The X-ray was obtained from one of the patients in the clinic.

From the first four to six months of the newborn's life, radiography is considered the primary investigation for assessing hip growth and maturation [13]. Radiography can be used to evaluate hip development and ossification, as well as to assess for avascular necrosis. Sharp's angle and the acetabular index are utilized to examine the acetabular anatomy, while the relationship between the acetabulum and the femoral head is assessed by Shenton's line. The Wiberg's center-edge angle and the proportion of the femoral head covered are employed to gauge the amount of concentrically reduced hips [14]. Shenton's line is also used to evaluate the relationship between the acetabulum and the femoral head (Figure 1).

Ultrasound

In infants younger than four months, US is preferred over radiography because the hip in this cohort is largely made of cartilage, which prevents effective visualization by radiography. With US, it is possible to clearly visualize the femoral head and where it lies in relation to the acetabulum. In addition, it allows us to measure the acetabular depth and the acetabular roof angle [15]. US can be done statically or dynamically. Static US examines the anatomy of the joint, whereas dynamic US examines the stability of the hip joint by conducting stress maneuvers while imaging the hip [15].

Management of DDH

The outcome of DDH management is highly dependent on the age of presentation and intervention; the higher the age, the more severe the outcome. The chances of successfully re-aligning the femoral head with the acetabulum significantly reduce after the age of 3-4 years, emphasizing the importance of early intervention. It is suggested that by the age of eight, the potential complications of treatment outweigh the benefits, making the outcomes no better than if DDH were left untreated [16,17].

Acetabular dysplasia correction

Repositioning the displaced hip (either dislocated or subluxated) into its normal position can act as a stimulus that promotes normal acetabular maturation. The Pavlik harness [18] is employed to stabilize the hip in infants with DDH who are less than six months old. The harness maintains the hip in a flexed and abducted position, in which the acetabulum and the femoral head are well aligned. Studies have indicated that infants with a positive Barlow's test have a higher success rate (more than 90% success rate) with the Pavlik harness, compared to infants with a positive Ortolani's test (21-37% failure rate) [19,20]. Some factors that increase the risk of Pavlik harness failure include intervention at greater than 7 weeks of age, being male, multi-gravida pregnancy, and foot deformities [20]. In infants with a positive Ortolani's test, one prognostic factor for harness failure is the degree of dysplasia on ultrasound; the more severe the dysplasia, the higher the risk of failure [19].

The patient’s age and acetabular index are used to provide insight into the likelihood of residual hip dysplasia in adulthood. For patients with residual hip dysplasia post-initial treatment, although there is no set time for performing acetabular and/or femoral osteotomies, they are necessary for this cohort of patients to reduce or prevent the risk of adult-onset coxarthrosis [18].

Although uncommon, certain complications can arise from the use of a Pavlik harness. These complications include femoral head avascular necrosis, anterior crural nerve palsy, and skin irritation [21]. Femoral nerve palsy becomes apparent when the infant ceases to exhibit spontaneous knee extension while wearing the harness. One study showed that all patients with anterior crural nerve palsy caused by the Pavlik harness experienced full recovery of nerve function after loosening or discontinuing the harness. Moreover, the study found that only 53% of patients with anterior crural nerve palsy, as a result of using the Pavlik harness, had successful treatment; 47% had unsuccessful treatment. Two possible explanations may contribute to this high treatment failure rate: either anterior crural nerve palsy occurs in more severe cases of DDH, or the occurrence of nerve palsy leads to termination of Pavlik harness use, which in turn results in higher treatment failure rates [21,22].

For patients aged 12-18 months with DDH or those less than one year old with an unsuccessful closed hip reduction, open hip reduction is recommended [23]. Recently, the primary focus of open reduction in DDH has shifted toward understanding and minimizing surgical complications. There are two approaches to performing open reduction: the anterior and the medial approach. The anterior approach is more traditionally used because it offers broader access to the acetabulum and enables capsulorrhaphy. Capsulorrhaphy is a surgical procedure that tightens the hip capsule and helps maintain hip stability; it can only be executed via the anterior approach. Conversely, the medial approach is less invasive and, unlike the anterior approach, does not involve splitting the iliac apophysis.

Surgeons favoring either the anterior or medial approach may argue that their chosen method has a lower risk of developing avascular necrosis; however, no research definitively supports one approach over the other regarding this risk. The choice usually depends on the surgeon's personal preference and the child's age. Avascular necrosis rates for both the anterior and medial approaches vary significantly from one study to another. Nonetheless, pooled meta-analysis data suggest an approximate 20% risk of avascular necrosis for both methods combined [23]. In patients older than two years, a surgical procedure called femoral shortening osteotomy can be incorporated into the open reduction surgery. This aims to reduce stress on the hip post-reduction and has shown effectiveness in reducing the risk of avascular necrosis and hip re-dislocation [23].

Pelvic osteotomies

In femoral osteotomies, the proximal femoral orientation is altered to a different position through de-rotation and increased varization of the hip. These changes aim to enhance hip stability and promote the maturation and development of the acetabulum [24]. This practice stems from the belief that hips in DDH typically exhibit elevated anteversion and varization, although this viewpoint is considered controversial. Anteversion of the femur is often considered the main factor contributing to the recurrence of hip subluxation; hence, a de-rotational osteotomy is crucial for achieving stable hip reduction. Studies have also shown that acetabular volume can be increased through varization [24].

Acetabular dysplasia is commonly characterized by a flattened and vertically oriented acetabulum. This condition results in the acetabulum not fully covering the femoral head upon hip reduction, or it may manifest as an increased risk of early onset hip arthritis. Pelvic osteotomies can be performed on patients who continue to experience hip dysplasia after initial treatment, to encourage normal development of the acetabulum. There is no definitive timeline for when to attempt pelvic osteotomies for residual dysplasia. However, it is suggested that before the age of 5, the acetabulum has the potential to reshape itself, and if well-aligned with the femoral head, the hip can develop normally [25]. Therefore, osteotomies are generally conducted between the ages of 3 and 5. Some of the most frequently employed osteotomy techniques are Dega, Salter, and Pemberton [25-28], named for the direction of the osteotomy. A review of recent literature indicates that these three techniques are equally effective in treating residual hip dysplasia [25,26].

**Figure 2:**
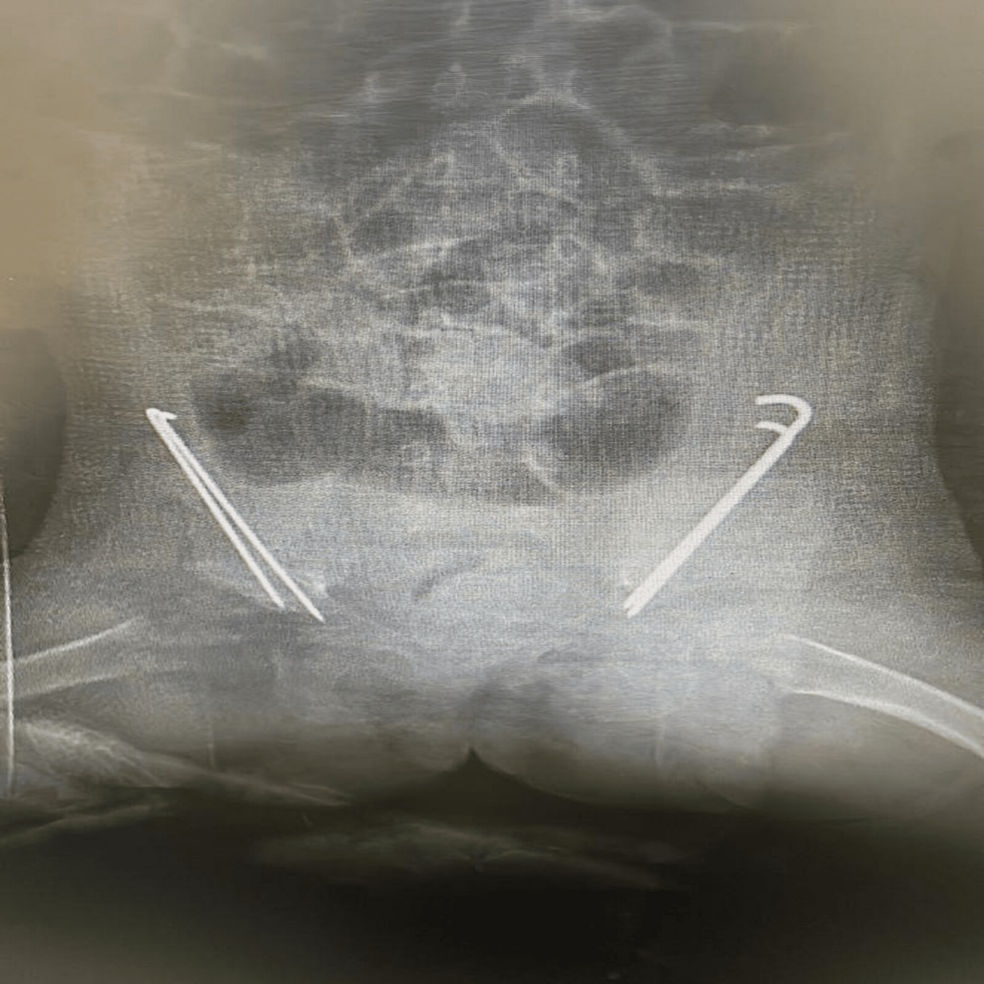
A four-year-old DDH patient who underwent femoral and pelvic osteotomy. The X-ray was obtained from one of the patients in the clinic.

## Conclusions

The outcome of DDH is dependent on the age of presentation, a more severe outcome is linked to a higher age of presentation. Treatment of DDH is challenging; however, the latest advancements in the field helped to improve our understanding of the disease, which resulted in advancements in the surveillance of the disease and the promotion of early intervention. In order to improve surveillance for DDH, Barlow’s and Ortalani’s tests should be added to the general neonatal examination universally as they can reliably detect DDH, whereas observing for asymmetrical thigh folds or clicks can be of an insignificant value. As for imaging, US of the hip as a universal screening tool is controversial, as it might result in overtreatment.
